# Esophageal Duplication Cyst: A Rare Cause of Esophagogastric Junction Obstruction

**DOI:** 10.1016/j.atssr.2025.09.003

**Published:** 2025-10-03

**Authors:** Johnny Wang, Ricardo A. Pascual, Ray Chihara, Min P. Kim

**Affiliations:** 1 Department of Surgery, Houston Methodist Hospital, Houston, Texas; 2 Department of Pathology and Genomic Medicine, Houston Methodist Hospital, Houston, Texas

## Abstract

Esophageal duplication cysts are rare benign esophageal masses. We present the case of a 37-year-old man with slowly progressing dysphagia and postprandial pain who was found to have a cystic mass measuring 4.6 cm × 5.4 cm × 4.6 cm above the esophagogastric junction. The manometry results were consistent with esophagogastric outflow obstruction. The patient underwent robot-assisted laparoscopic parasophageal mass resection with intraoperative endoluminal functional lumen imaging probe analysis. This report describes esophageal duplication cysts as a cause of esophagogastric junction obstruction and discusses a management approach for this condition.

Esophageal duplication cysts are rare congenital abnormalities secondary to failure of tubulation of the primitive esophagus. These cysts are usually asymptomatic in adults, although they can manifest with various symptoms, depending on their location. Here we present a case of an esophageal duplication cyst causing dysphagia and the surgical management of this condition with the aid of intraoperative endoluminal functional lumen imaging probe (EndoFLIP, Medtronic).

A 37-year-old man presented with slowly progressing dysphagia and postprandial epigastric pain. The patient had a previous diagnosis of gastroesophageal reflux disease from his primary care physician and was started on acid suppression medication. During the workup for coronavirus disease 2019 infection, the patient was incidentally found to have a 4.2 cm × 3.9 cm mass abutting the distal esophagus on computed tomographic angiography of the chest (Figures 1A, 1B). The patient underwent an endoscopic ultrasound (EUS) examination that showed a homogeneous, hypoechoic lesion 39 cm away from the incisors. The endoscopist performed a biopsy of the cyst that was not diagnostic. Magnetic resonance imaging revealed a well-circumscribed mass that appeared dark on T1 and bright on T2 images (Figures 1C, 1D). In addition, the patient underwent manometry, which showed an integrated relaxation pressure of 15.2 mm Hg and a distal contractile integral of 1547.1 mm Hg/s/-cm with 100% intact esophageal motility, consistent with esophagogastric junction outflow obstruction (EGJOO). A 24-hour impedance test revealed a total acid exposure time of 1.4% and a DeMeester score of 5.3, findings suggesting that the patient did not have reflux disease.

**Figure 1:**
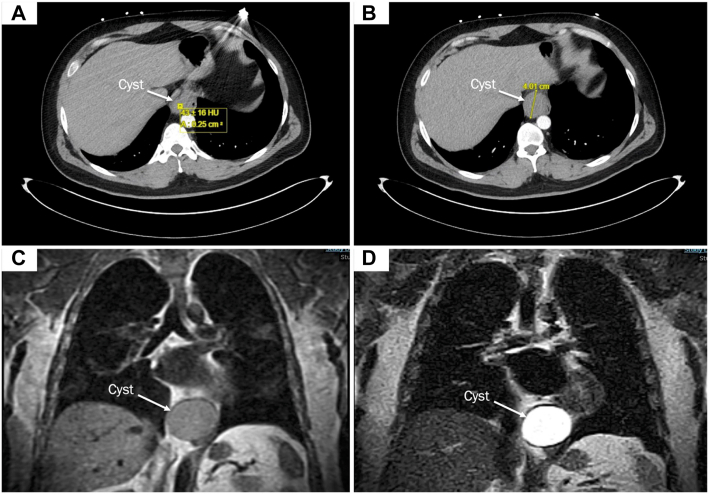
Imaging of the mass. Computed tomographic angiogram showing a paraesophageal mass with (A) 43 Hounsfield units (HU) and (B) measuring 4 cm. On magnetic resonance imaging, the mass appears (C) dark on T1-weighted imaging and (D) bright on T2-weighted imaging (D), findings suggesting that the mass is fluid filled.

The patient’s presenting symptoms of pain and dysphagia were likely secondary to EGJOO from the mass, with an erroneous diagnosis of gastroesophageal reflux disease previously. Although the cystic mass was likely the main driver of the obstruction, there was a possibility that an underlying motility disorder was also contributing to the EGJOO. The patient was recommended to undergo robotic-assisted laparoscopic resection of the mass with intraoperative endoluminal functional lumen imaging probe analysis (EndoFLIP) to assess whether the patient had a concurrent motility disorder. EndoFLIP is a technology that allows measurement of distensibility at a certain location in the gastrointestinal tract such as the EGJ. The balloon catheter is placed at the EGJ, and the balloon is filled with conductive fluid. Sensors in the balloon measure the minimal diameter (Dmin), cross-sectional area, and pressure at that location. The cross-sectional area divided by the pressure yields the distensibility index (DI), which provides a reference point that can guide further intraoperative management. At the start of the case, intraoperative EndoFLIP analysis showed a Dmin of 5 mm and a DI of 0.2 mm^2^/mm Hg, with 30 cc in the balloon (Figure 2C). Using robotic technology, we mobilized the esophagogastric junction (EGJ), which showed a large cystic mass abutting the distal esophagus (Figure 2A). After mobilizing the cyst from the right crus and diaphragm, the cyst was drained to aid in dissection, revealing thick green fluid. The cyst capsule was then dissected from the smooth muscle layers and the mucosa of the esophagus (Figure 2B). After removal of the cyst, the muscular layers were approximated and closed with a 3-0 V-Loc absorbable suture (Medtronic). Intraoperative EndoFLIP analysis was repeated after cyst resection, and it showed a Dmin of 12.2 mm and a DI of 3 mm^2^/mm Hg, findings suggesting resolution of the EGJOO (Figure 2D). The diagnosis of motility disorder was excluded. The crura of the diaphragm were then closed with a 2-0 V-Loc nonabsorbable suture, with a final DI of 1.5 mm^2^/mm Hg after closure (Figure 2E). Because the final DI was 1.5 mm^2^/mm Hg, fundoplication was not performed.

**Figure 2:**
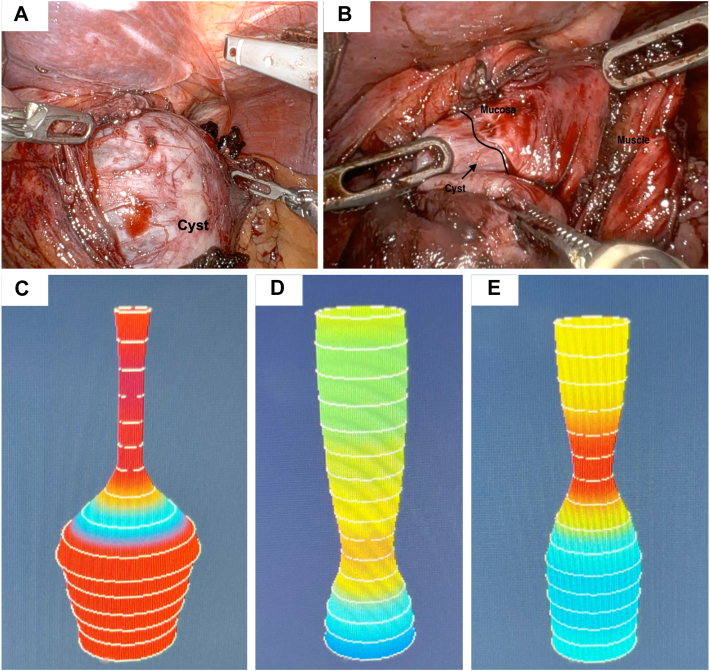
Intraoperative photographs show (A) a large paraesophageal cyst visualized and (B) dissected from the esophagus. EndoFLIP (Medtronic) topography with the balloon inflated to 30 cc demonstrating (C) a distensibility index of 0.2 mm^2^/mm Hg before cyst resection, (D) a distensibility index of 3 mm^2^/mm Hg after cyst resection, and (E) a distensibility index of 1.5 mm^2^/mm Hg after closure of the crura.

The patient was discharged on postoperative day 2 on a full-liquid diet. The patient returned to the clinic 5 weeks later, was tolerating a regular diet, and reported complete resolution of dysphagia and reflux-like symptoms. The final pathologic findings were consistent with an esophageal duplication cyst with ciliated epithelium (Figure 3).

**Figure 3:**
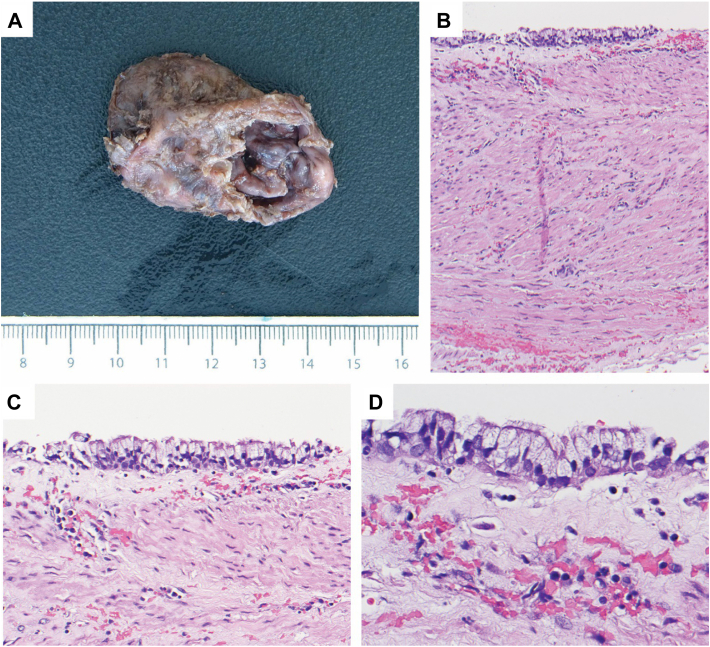
Final pathologic findings. (A) Gross photograph of the paraesophageal mass shows a collapsed cyst with a tan-brown mucosal surface. (B) Low magnification shows that the cyst wall consists of two smooth muscle layers with a partially intact epithelial lining. (hematoxylin and eosin stain; Original magnification ×100.) (C, D) Higher magnifications show that the cyst is lined by ciliated columnar epithelium, and the stroma shows a mixed chronic inflammatory infiltrate. (C, hematoxylin and eosin stain; Original magnification ×200; D, hematoxylin and eosin stain; original magnification ×400.)

## Comment

Duplication cysts can occur anywhere along the alimentary tract, with the ileum the most common site and the esophagus the second most common location, comprising 10% to 15% of all gastrointestinal duplications.^1^ Duplications can be cystic or tubular, and cystic is more prevalent. Although typically diagnosed in childhood, esophageal duplication cysts can manifest in adults with a variety of symptoms, including chest pain, respiratory distress, hematemesis, and dysphagia, depending on the location of the cyst.^1^^,^^2^

In patients without symptoms, diagnostic cross-sectional imaging of the chest with esophagogastroduodenoscopy and EUS is usually indicated. Although our patients underwent an EUS examination with biopsy at an outside institution, this is not recommended because it can potentially seed infection.^3^^,^^4^ Complete resection of the cystic mass is the treatment of choice for symptomatic patients, and this procedure may be best performed at an experienced center.

Given the location of the mass at the EGJ, we performed robotic-assisted laparoscopic resection. We used intraoperative EndoFLIP analysis to guide our treatment and measure the treatment response. EndoFLIP is a technology that uses impedance planimetry to assess luminal distensibility during balloon distension. This technology has been very useful in the diagnosis of gastrointestinal motility disorders such as achalasia and during hiatal hernia repair with fundoplication.^5^ Our patient had an elevated integrated relaxation pressure found on manometry; however, it was unclear whether this was secondary to the mechanical obstruction of the cyst on the esophagus alone or an additional underlying motility disorder. EndoFLIP analysis was performed at 3 points during the operation: (1) before mass resection to establish a baseline, (2) after mass resection to assess treatment response, and (3) after closure of the crura to assess whether fundoplication was needed. EndoFLIP provides information at the time of surgery to help surgeons tailor the operation.^5^ Typically, patients with a tight EGJ needing a Heller myotomy have a DI of <0.8, whereas patients with hiatal hernia have a DI >2.3.^5^ Because our patient’s DI of 1.5 mm^2^/mm Hg after crura closure was between the 2 values and the patient’s symptoms were not related to reflux, we opted not to perform a Toupet fundoplication.

In conclusion, we describe a case of intramural esophageal duplication cyst manifesting as dysphagia that was treated with robotic-assisted laparoscopic resection with the aid of intraoperative EndoFLIP analysis. EndoFLIP appears to be a helpful technology that can provide real-time feedback on treatment response and guide further intraoperative decision making during resection of paraesophageal masses located at the EGJ.
